# 

**Published:** 1870-01

**Authors:** 


### *CONTENTS:*

Original Communications:
Art. I. Brief Review of the Natural Supports of the Uterus. By H. W. Jones, M.D., - - 1
" II. On Life, from a Physical Basis. By Z. C. McElroy, M.D., 6
" III. Cases in Surgery—Abscess in Long Bones. By H. O. Hitchcock, A.M., M.D., - 18
" IV. Venereal Excess, with Mental Impairment. By J. S. Whitmire, M.D., - - - - 23
" V. Tænia Solium. By D. C. Daviess, M.D., - - - - 27
" VI. A Case of Placenta Prævia. By I. B. Washburn, M.D., - 29
" VII. A Case of Poisoning with Sulphate of Copper. By Dr. D. B. Hillis, - - - - - 30
" VIII. Death from Strangulation of a portion of the Ileum. By Wm. S. Lincoln, M.D., - 32

Correspondence:
Glycerine and Carbolic Acid for Burns. By T. Williams, M.D., - - - - - 34
Imaginary Cases, - - - - - - - - 35

Hospital Reports:
Art. I. Cook County Hospital. By Curtis T. Fenn, M.D., - - - - - -

Original Translations:
Art. I. Clinical Lectures upon the Mental and Nervous Diseases. By M. Magnan, Paris, (Concluded,) - - - 38

Editor's Book Table:
Vogel on Children, - - - - - - - 47
Smith's Wasting of Children, - - - - 48
Bartholow's Hypodermic Medication, - 49
Pamphlets, - - - - - - - - - - - 50

Editorial:
A Word about Hospitals, - - - - - 61

Obituary:
Death of Dr. J. R. Hamill, - - - - 64

